# Intensified blood pressure control during hospital admission and on discharge: a systematic review and meta-analysis of retrospective cohort studies

**DOI:** 10.3389/fcvm.2026.1691926

**Published:** 2026-02-05

**Authors:** Yevhen Kushnir, Nelson Barrera, Pedro Arias-Sanchez, Erick Romero, Iurii Statnii, Anton Stolear, Kristina Golovataya, Maria Fernanda Solorzano, Salim Baghdadi, Evgeny Shkolnik

**Affiliations:** 1 SBH Health System, Department of Internal Medicine, City University of New York School of Medicine, Bronx, NY, United States; 2 Department of Cardiovascular Diseases, Yale Bridgeport Hospital, Bridgeport, CT, United States; 3 Yale University School of Medicine, New Haven, CT, United States; 4 McLaren Greater Lansing, Department of Internal Medicine, Lansing, MI, United States

**Keywords:** blood pressure, intensified management, conservative management, non-cardiac hospitalizations, inpatient hypertension

## Abstract

**Introduction:**

Limited single-center studies suggest that intensified blood pressure (BP) control in patients with asymptomatic elevated BP during non-cardiac admissions may lead to worse outcomes. In this study, we performed a systematic review and meta-analysis exploring the safety of intensified BP control vs. a more conservative approach in patients with asymptomatic elevated BP during non- cardiac admissions and at discharge, focusing on stroke, acute kidney injury (AKI), myocardial infarction (MI), and length of stay (LOS).

**Methods:**

Four retrospective propensity score-matched cohort studies (*n* = 77,448) were included. The intensified BP control group (*n* = 38,724) received newly initiated, increased dose, intravenous (IV), or *pro re nata* (PRN) antihypertensive medication, including PRN with scheduled therapy. The non-intensified group (*n* = 38,724) included patients continuing their preadmission regimen, scheduled, or with no PRN antihypertensives. Follow-up began after the first inpatient antihypertensivedose or at discharge and continued until (1) 30 days postdischarge, (2) hospitaldischarge, or (3) both, depending on the study. Patients with hypertensive emergencies, stroke, MI, or aortic dissection at admission were excluded.

**Results:**

Intensified BP control was associated with increased odds of stroke (OR 3.77; 95% CI 1.38–10.27; *p* < 0.010), AKI (OR 1.23; 95% CI 1.13–1.33; *p* < 0.00001), and longer LOS (MD 1.17; 95% CI 1.11–1.93; *p* < 0.00001). No statistically significant increase of MI was noted (OR 2.04; 95% CI 0.85–4.89, *p* = 0.11). Intensified BP control during non-cardiac hospitalizations and at discharge was linked to higher odds of stroke, AKI, and prolonged hospitalization.

**Conclusions:**

A more conservative approach may be safer in the absence of acute indications for BP lowering. Prospective, randomized inpatient BP trials, particularly those distinguishing interventions initiated during hospitalization vs. at discharge are warranted to clarify causal relationships and guide evidence-based inpatient BP management.

**Systematic Review Registration:**

https://www.crd.york.ac.uk/PROSPERO/view/566609, identifier CRD42024566609.

## 1 Introduction

Hypertension affects approximately 50% of individuals aged over 20 years and remains a significant risk factor for cardiovascular diseases, stroke, and mortality (1, 2). Among hospitalized patients, the prevalence of hypertension is particularly high, ranging from 50.5% to 72% (3). The intensification of antihypertensive treatment during hospitalization has been inconsistent, resulting in 37%–77% of hypertensive patients remaining hypertensive at the time of discharge (3). Furthermore, many patients with inpatient hypertension continue to have uncontrolled BP during outpatient follow-up (3), underscoring the need for improved management strategies. However, changes in medication regimens may put patients at higher risk of adverse events (4). A study in an emergency department found that inpatient blood pressure readings can differ significantly from the patient's home blood pressure level (5). Moreover, patients discharged on substantially intensified antihypertensive regimens have been reported to experience hypotension during follow-up visits (5).

The 2017 and 2019 American College of Cardiology/American Heart Association/guidelines do not recommend a specific approach for asymptomatic patients with elevated blood pressure in hospitalized settings (6, 7). However, subsequent observational studies suggest that more intensified blood pressure control may be associated with adverse outcomes (3, 8). The 2024 American Heart Association scientific statement consolidates findings from previous studies and recommends that treatment be reserved for select cases, emphasizing observation in the absence of symptoms, noting the need for further studies to clarify whether clinical benefits exist for asymptomatic patients with markedly elevated blood pressure (9). Because of the ongoing lack of randomized controlled trials with data regarding the risks and benefits of blood pressure control in asymptomatic patients, we conducted a systematic review and meta-analysis to evaluate the safety of intensified BP control during non-cardiac hospitalizations and at discharge in patients with asymptomatic elevated BP.

## 2 Methods

### 2.1 Eligibility criteria

We considered studies eligible for inclusion if they (1) were randomized controlled trials (RCTs) or non-randomized cohorts, (2) compared intensified vs. non-intensified management of elevated blood pressure during hospital admission OR on discharge, (3) patients with non-cardiac admissions.

The intensified BP control group was defined as patients receiving a newly initiated, increased dose, intravenous (IV), or *pro re nata* (PRN) antihypertensive medication, including PRN combined with scheduled therapy. The non-intensified blood pressure control group was defined as patients who continued on their preadmission regimen, scheduled, or had no PRN antihypertensive medication in the regimen. Non-cardiac admission was defined as conditions that do not require specific blood pressure control goals and regimens (studies with patients who had a hypertensive emergency, stroke, MI, or aortic dissection on admission/during hospitalization were excluded).

Patient follow-up began either after the first inpatient dose of antihypertensive medication or at discharge and continued until (1) 30 days postdischarge, OR (2) hospital discharge, OR (3) hospital stay and 30 days’ postdischarge, depending on the study design. Studies were excluded if they (1) lacked a control group or (2) involved a cardiac cause of admission. Definitions of intensified blood pressure control varied slightly across studies and are detailed in Table 1.

**Table 1:** Baseline characteristics of the included studies.

Study, year	Patients, n	Blood pressure control criteria	Follow-up period	When intensification occurred	Age,^b^ years	Male, %	Previous stroke, I-BP/nI-BP	HTN, %	SBP on admission, mm Hg, mean	Previous MI, %	Arrhythmias	CKD, %
	i-BP/nI-BP	I-BP/nI-BP			I-BP/nI-BP	I-BP/nI-BP		I-BP/nI-BP	I-BP/nI-BP	I-BP/nI-BP	I-BP/nI-BP	I-BP/nI-BP
Anderson, 2019 (4)	2,028/2,028	New or higher-dose anti-HTN^a^/before admission regimen	30 days after discharge	At discharge	76.8/76.6	97.5/97.8	17.4./18.6	NA	138.3/137.9	5.5/5	32/31.2	NA
Rastogi, 2020 (8)	4,520/4,520	IV anti-HTN^a^ medication or new class of oral anti-HTN^a^/before admission regimen	Hospitalization and 30 days after discharge	On admission	69.8/69.7	44/43.3	7.2/7.2	53/52.4	138.1/138.1	1.8/1.7	12.3/11.8	14.3/14.7
Mohandas, 2021(26)	4,219/4,219	PRN^b^BP + Scheduled BP/Scheduled BP	Hospitalization	On admission	62 ± 16/ 63 ± 15	45/45	33/34	90/89	157/156	15/15	N/A	40/41
Caneles, 2025(27)	27,957/27,957	PRN^b^BP/No PRN BP	Hospitalization	On admission	71.0 ± 11.8/71.4 ± 11.5	96.7/96.8	12.8/12.8	76.8/76.7	155.7/154.7	N/A	N/A	26.3/27.6

CKD, chronic kidney disease; HTN, hypertension; I-BP, intensified blood pressure group; MI, myocardial infarction; N/A, not available; nI-BP, non-intensified blood pressure group; SBP, systolic blood pressure.

a Antihypertensive medication.

b As needed.

### 2.2 Data source and search strategy

We systematically searched PubMed, Embase, and Cochrane from inception to January 2025 using the terms “inpatient blood pressure control outcomes” and (Hospitalized OR Inpatient OR Non-cardiac) AND (Blood pressure OR Hypertension OR Antihypertensive medication OR Blood pressure control) AND (Intensified OR Conservative OR As needed OR PRN OR Intravenous antihypertensive) AND (Stroke OR Acute Kidney Injury OR AKI OR Myocardial Infarction OR MI OR Length of Stay OR Hospital stay OR Mortality). In addition, we manually searched the references from all included studies for additional studies. Two authors (YK and NB) independently screened titles and abstracts and fully evaluated the articles for eligibility based on prespecified criteria. Discrepancies were resolved in a panel discussion between authors. The prospective meta-analysis protocol was registered at the International Prospective Register of Systematic Reviews (PROSPERO; #CRD42024566609) in July 2024.

### 2.3 Endpoints, meta-regression, and sensitivity analysis

The outcomes of interest were acute kidney injury (AKI), stroke, myocardial infarction (MI), and length of stay (LOS). We performed a sensitivity analysis for studies in which blood pressure (BP) intensification occurred only during hospital stay to isolate the effect of BP intensification during hospitalization. Between-study heterogeneity was investigated using random-effects meta-regression (restricted maximum likelihood estimator with Hartung–Knapp adjustment), testing the effect of mean age, baseline systolic blood pressure (SBP), and comorbidities (% hypertension, stroke, and CKD) on the pooled effect size. In addition, we performed a leave-one-out sensitivity analysis to explore the impact of separate studies on the cumulative analysis for stroke and AKI.

### 2.4 Quality assessment

Non-randomized studies were assessed using the Risk of Bias in Non-randomized Studies of Interventions tool (ROBINS-I) (10). Two independent authors (YK and NB) conducted the risk of bias assessment. Disagreements were resolved through consensus after discussing the reasons for the discrepancies. Publication bias was evaluated using a funnel-plot analysis of point estimates about study size.

### 2.5 Statistical analysis

This systematic review and meta-analysis was performed and reported by following the Cochrane Collaboration Handbook for Systematic Reviews of Interventions and the Preferred Reporting Items for Systematic Reviews and Meta-Analysis (PRISMA) Statement guide (11). Odds ratios (ORs) with 95% confidence intervals (CIs) were used to compare treatment effects for categorical endpoints. Continuous outcomes were compared using standardized mean differences (MD). Heterogeneity was assessed using I^2^ statistics and the Cochran Q test; *p*-values <0.10 and *I*^2^ > 25% were considered indicative of significant heterogeneity. We applied the random-effects model with the Restricted Maximum-Likelihood (REML) estimator. A statistical analysis was performed using ReviewManager (Cochrane Center, The Cochrane Collaboration, Denmark) and R (4.5.1 version).

## 3 Results

### 3.1 Study selection and baseline characteristics

**Figure 1:**
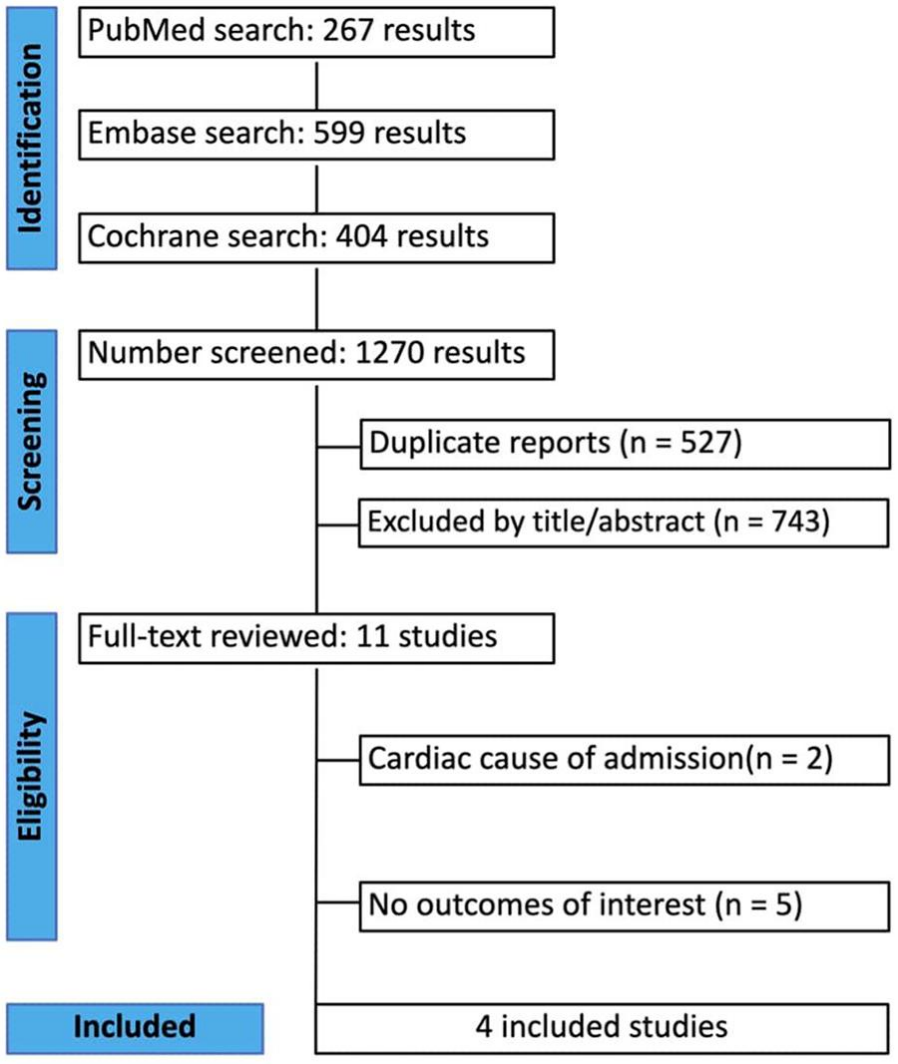
A PRISMA flow diagram of study screening and selection.

Our systematic search yielded 1,270 results. After removing duplicate records and ineligible studies, 11 studies remained and were fully reviewed based on the inclusion criteria. Of these, four retrospective propensity score–matched cohort studies, comprising 77,448 patients, were included. Comprehensive details of the study selection are given in Figure 1.

**Figure 2:**
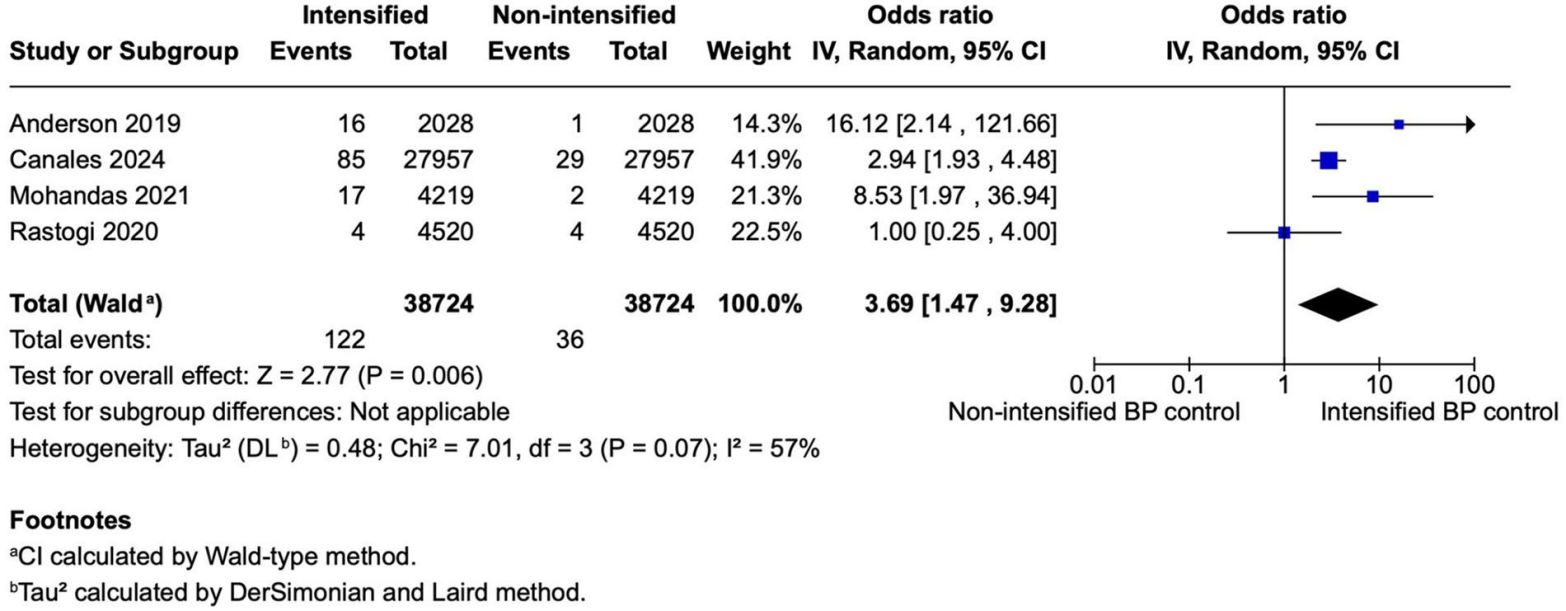
A forest plot showing the odds of stroke between intensified and non-intensified inpatient blood pressure control strategies. Each study is represented by a blue square, proportional to its weight in the random-effects model, and the horizontal lines indicate the 95% CI. The pooled OR is shown as a black diamond. A random-effects model (restricted maximum-likelihood method) was applied with confidence intervals calculated using the Wald-type method. Overall, intensified BP control was associated with a significantly higher odds of adverse events compared with non-intensified control (pooled OR = 3.77, 95% CI 1.38–10.27, *p* = 0.010; *I*^2^ = 63%). BP, blood pressure; CI, confidence interval; IV, inverse variance; OR, odds ratio; REML, restricted maximum-likelihood; Wald, Wald-type method.

**Figure 3:**
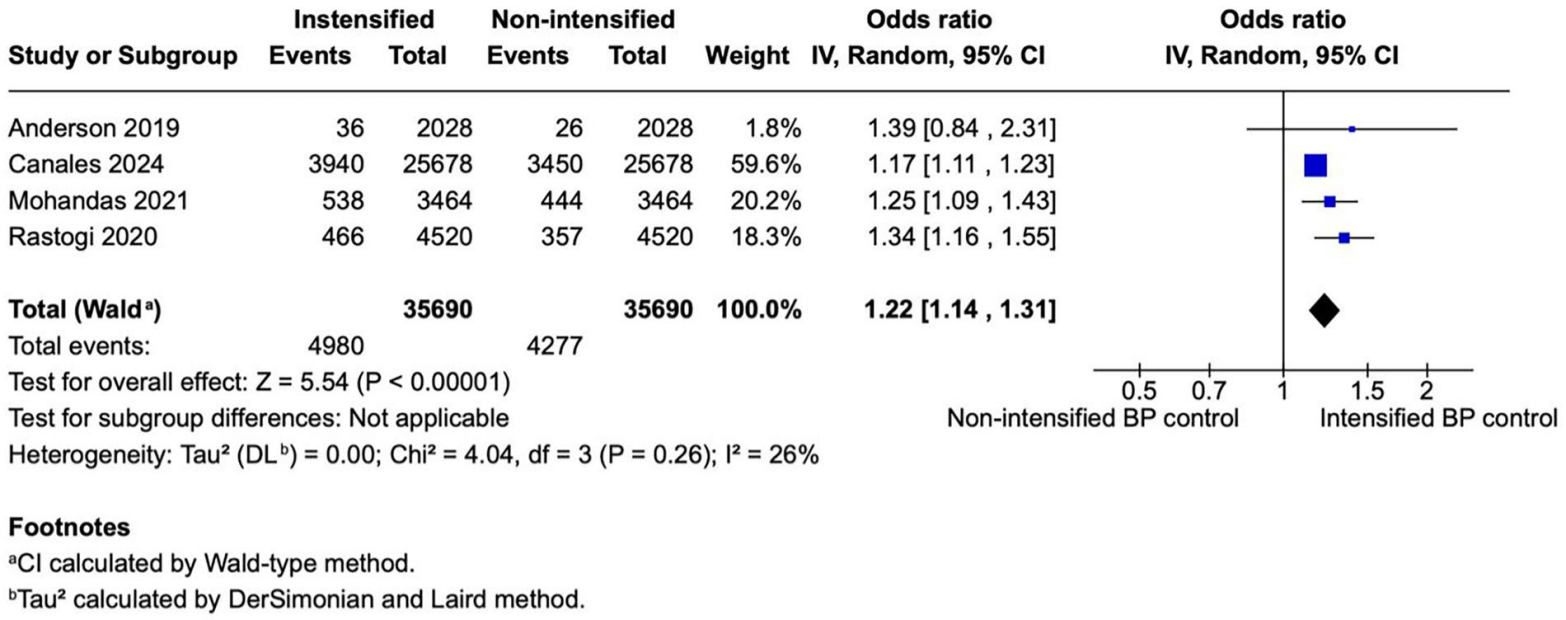
A forest plot showing the odds of AKI between intensified and non-intensified inpatient BP control strategies. Individual study estimates are represented by blue squares proportional to study weight, with the horizontal lines denoting 95% CIs. The pooled effect estimate (black diamond) was calculated using a random-effects model based on the REML method, with CIs derived using the Wald-type approach. Overall, intensified BP control was associated with a modest but statistically significant increase in the odds of adverse events compared with non-intensified control (pooled OR = 1.23, 95% CI 1.13–1.33; *p* < 0.00001). Between-study heterogeneity was moderate (*I*^2^ = 38%). AKI, acute kidney injury; BP, blood pressure; CI, confidence interval; IV, inverse variance; OR, odds ratio; REML, restricted maximum-likelihood; Wald, Wald-type method.

**Figure 4:**
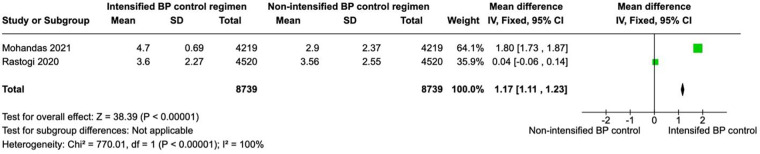
A forest plot showing the length of stay in intensified and non-intensified blood pressure control groups. Each study is represented by a green square proportional to its weight, with the horizontal lines indicating 95% CIs. The pooled mean difference (black diamond) was calculated using a fixed-effects inverse-variance model. Overall, patients in the intensified BP control group had a significantly higher length of stay SBP compared with those in the non-intensified group (pooled mean difference = 1.17 mm Hg, 95% CI 1.11–1.23; *p* < 0.00001). Heterogeneity across studies was high (*I*^2^ = 100%, *p* < 0.00001). BP, blood pressure; CI, confidence interval; IV, inverse variance.

**Figure 5:**
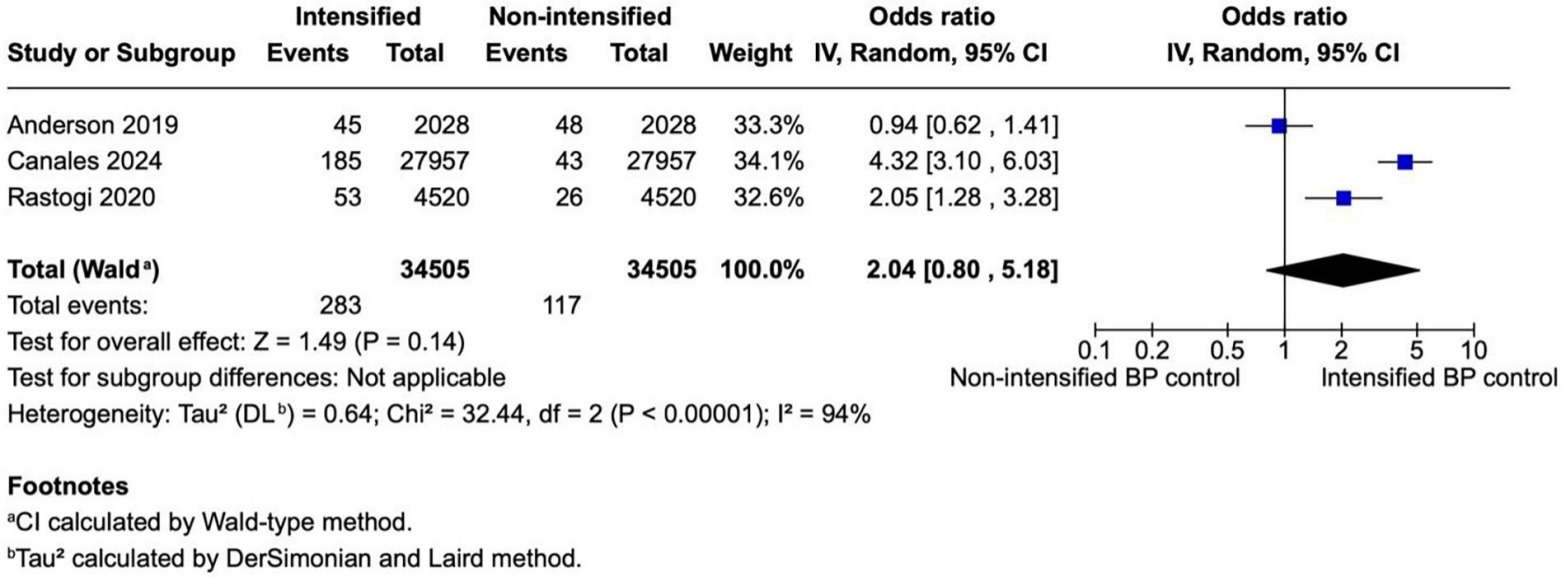
A forest plot showing the odds of myocardial infarction in intensified and non-intensified blood pressure control groups. Individual studies are represented by blue squares proportional to their weight, with the horizontal lines indicating 95% CIs. The pooled estimate (black diamond) was calculated using a random-effects model (restricted maximum-likelihood method) with CIs derived by the Wald-type approach. Across three retrospective cohort studies, intensified BP control was associated with a non-significant trend toward higher odds of MI event (pooled OR = 2.04, 95% CI 0.85–4.89; *p* = 0.11). Substantial between-study heterogeneity was observed (*I*^2^ = 93%, *p* < 0.00001). BP, blood pressure; CI, confidence interval; IV, inverse variance; MI, myocardial infarction; OR, odds ratio; REML, restricted maximum-likelihood; Wald, Wald-type method.

A total of 38,724 patients received an intensified blood pressure control regimen, while 38,724 patients received a non-intensified blood pressure control regimen. The mean SBP in the intensified group was 147.3 mmHg, compared with 146.7 mmHg in the non-intensified group. The mean age was 69.9 years in the intensified management arm and 70.1 years in the conservative arm. In the intensified group, 70.8% of patients were male, while 70.7% were male in the non-intensified group. Table 1 summarizes the main characteristics of the included studies. Outcome-level sample sizes varied across studies and are mentioned in the pooled analysis section and Figures 2–5 for each specific outcome.

### 3.2 Pooled analysis of all studies

#### 3.2.1 Incidence of stroke

Four studies (*N* = 77,448) reported stroke outcomes, including 38,724 patients per group. The pooled analysis showed significantly higher odds of stroke in the intensified blood pressure control group compared with the non-intensified control group (OR 3.77; 95% CI 1.38–10.27; *p* < 0.010; Figure 2). Heterogeneity was moderate, with *I*^2^ = 63%.

#### 3.2.2 Incidence of AKI

Four studies (*N* = 71,380) reported AKI outcomes, including 35,690 patients per group. The pooled analysis showed significantly higher odds of AKI in the intensified blood pressure control group compared with the non-intensified control group (OR 1.23; 95% CI 1.13–1.33; *p* < 0.00001; Figure 3). Heterogeneity was low, with *I*^2^ = 38%.

#### 3.2.3 Length of stay

Two studies (*N* = 17,478) reported hospital length of stay, with 8,739 patients in each group. Intensified blood pressure control was associated with a significantly longer length of stay than non-intensified blood pressure control (MD 1.17; 95% CI 1.11–1.23; *p* < 0.01; Figure 4). The results showed a high heterogeneity with *I*^2^ = 100%.

#### 3.2.4 Myocardial infarction

Three studies (*N* = 69,010) reported MI outcomes, including 34,505 patients per group. There was no statistically significant difference in the odds of myocardial infarction between the intensified blood pressure control group and the non-intensified blood pressure control group (OR 2.04; 95% CI 0.85–4.89; *p* = 0.11; Figure 5). The results showed a high heterogeneity with *I*^2^ = 9%.

#### 3.2.5 Meta-regression

To explore sources of between-study heterogeneity, we used univariable random-effects meta-regression models (Hartung–Knapp adjustment). As shown in Table 2, mean age was significantly associated with the pooled effect size (*β* = 0.089, 95% CI 0.043–0.136; *p* = 0.014). Each additional year of mean age corresponded to an approximately 9% increase in the odds of adverse outcomes, fully explaining between-study heterogeneity (*R*^2^ = 100%).

**Table 2:** Univariable meta-regression results (Hartung–Knapp REML models).

Moderator	*β* (log OR)	95% CI	*p*-value	Exp (*β*) (OR)	*R*^2^ (%)	Direction of association
Mean age (years)	0.089	0.043–0.136	0.014	1.09 per +1 year	100	Older cohorts → higher risk
SBP (mm Hg)	–0.039	–0.159–0.082	0.30	0.96 per +1 mm Hg	31	Non-significant, slight negative trend
Hypertension (% per 10 points)	–0.246	–2.71–2.22	0.43	0.78 per +10%	42	Non-significant
Prior stroke (% per 10 points)	–0.412	–2.84–2.02	0.28	0.66 per +10%	86	Non-significant
CKD (% per 10 points)	–0.421	–2.62–1.78	0.25	0.66 per +10%	92	Non-significant

Random-effects meta-regression analyses were performed using the Hartung–Knapp–Sidik–Jonkman adjustment and restricted maximum-likelihood estimation to explore potential study-level modifiers of the pooled odds ratio. Among evaluated moderators, mean age was significantly associated with the overall effect size (*β* = 0.089, 95% CI 0.043–0.136; *p* = 0.014), indicating that each additional year of mean age corresponded to an approximately 9% increase in the odds of adverse outcomes and accounted for all between-study heterogeneity (*R*^2^ = 100%). Systolic blood pressure on admission, prevalence of hypertension, prior stroke, and chronic kidney disease showed no statistically significant associations (all *p* > 0.05).

In contrast, other study-level characteristics—including admission SBP (*β* = –0.039, *p* = 0.30), prevalence of hypertension (*β* = –0.246, *p* = 0.43), prior stroke (*β* = –0.412, *p* = 0.28), and chronic kidney disease (*β* = –0.421, *p* = 0.25)—were not significantly associated with the pooled effect size. These non-significant trends suggested a possible but weak inverse relationship between higher comorbidity burden and outcome, with minimal explanatory contribution to heterogeneity (*R*^2^ = 31%–92%).

#### 3.2.6 Sensitivity analyses

Sensitivity analyses restricted to studies with BP intensification during hospitalization revealed a statistically significant association with MI (OR 3.08; 95% CI 1.46–6.30; *p* = 0.003; *I*^2^ = 84%; Supplementary Figure S3). A similar result to a pooled analysis was yielded for stroke (OR 2.88; 95% CI 1.16–7.16; *p* = 0.02; *I*^2^ = 55%; Supplementary Figure S1) and AKI (OR 1.22; 95% CI 1.13–1.33; *p* < 0.00001; *I*^2^ = 47%; Supplementary Figure S2).

A leave-one-out sensitivity analysis was performed to explore the impact of separate studies on the cumulative analysis for stroke and AKI. Overall, the results remained similar to those of the primary analysis. The sensitivity analysis results are presented in Supplementary Figures S4, S5.

#### 3.2.7 Quality assessment

No studies were considered at high risk of bias as described in Supplementary Table S1. In the funnel plot (Supplementary Figure S6), studies occupied symmetrical distribution according to weight and converged toward the pool effect as the weight increase.

## 4 Discussion

This systematic review and meta-analysis of four studies involving 77,448 patients evaluated the clinical outcomes associated with intensified vs. non-intensified blood pressure (BP) management at admission and discharge in asymptomatic patients with elevated BP. Intensified BP control was associated with a significantly higher odds of stroke (OR 3.77), AKI (OR 1.23), and a longer hospital stay (MD 1.17 days). While the pooled analysis did not show a statistically significant increase in myocardial infarction (MI), a sensitivity analysis limited to in-hospital BP intensification demonstrated a significant association. This analysis addresses a critical gap in inpatient hypertension management, where evidence-based guidance remains limited.

Asymptomatic elevated blood pressure (BP) was reported to be present in 50%–72% of hospitalizations (3). In patients with hypertensive emergency, the approach to blood pressure control is well established; however, a majority of hospital encounters with elevated blood pressure do not have an indication to pursue tight blood pressure control. The 2019 American Heart Association guideline does not recommend a specific approach because of the lack of evidence (7); however, the 2024 AHA Scientific Statement recommends observation while highlighting that further studies are needed to clarify whether there is a clinical benefit for asymptomatic patients with markedly elevated blood pressure (9).

Elevated BP readings can be a concerning finding during a hospital stay. A survey of 181 residents across three US hospitals revealed that 44% would initiate or adjust antihypertensive treatment if the SBP ranged from 140 to 159 mmHg (12). In clinical practice, elevated BP readings often prompt PRN antihypertensive administration, driven by cardiovascular risk profiles, nursing alarms, and vital sign thresholds (9). This reactive approach can induce BP variability by overcorrection and withholding of scheduled antihypertensives, potentially creating a self-perpetuating cycle.

The hospital environment is teeming with reversible causes for elevated BP. Key contributors include anxiety, sleep deprivation, psychological stress, and pain, all of which need to be carefully assessed and managed. Various inpatient medications such as IV fluids, corticosteroids, and NSAIDs can also elevate BP. Moreover, inaccurate home medication reconciliation can lead to rebound hypertension. A retrospective review found that 41% of patients prescribed PRN antihypertensive medications did not receive their usual home BP regimen while hospitalized. Addressing these factors is crucial for effective BP management and preventing unnecessary complications (13).

### 4.1 Mechanisms

Autoregulation is a mechanism that enables organs such as the brain, heart, and kidneys to maintain stable blood perfusion by adjusting their vasculature resistance in response to fluctuations in perfusion pressure (14). In individuals with normal blood pressure, the brain's vascular resistance adjusts over a wide range of mean arterial pressures to maintain consistent blood flow (15). However, in patients with chronically elevated blood pressure, the autoregulatory curve shifts to the right, with a subsequent risk of hypoperfusion when administering antihypertensive medication, as the precise lower limit of autoregulation in individual hypertensive patients is often unknown (15). This shift in autoregulation could help explain the study's findings, where intensified blood pressure control was associated with significantly higher odds of stroke and AKI. Meta-regression identified mean age as a significant effect modifier, suggesting that older inpatient populations derive less benefit—or greater harm—from intensified BP control. This aligns with evidence that age-related vascular stiffness and autonomic dysregulation may reduce tolerance for rapid BP lowering (16).

### 4.2 Stroke

Elevated BP is a well-established and highly impactful modifiable risk factor for stroke (17). The slight difference in mean SBP between groups (147.3 vs. 146.7 mmHg) seems insufficient to explain the observed adverse outcomes. However, mean values may obscure clinically significant fluctuations in BP. BP variability, not captured in these averages, could be a more important driver of stroke risk. Given the rightward shift in the autoregulatory curve among chronically hypertensive patients, intensified BP lowering may reduce perfusion below the autoregulatory threshold, increasing stroke risk. Intensified BP control can lead to substantial BP fluctuations, which might be more harmful than consistently elevated BP (18). Higher BP fluctuations after stroke are associated with a higher risk of death within 90 days (19). Paradoxically, while efforts to control BP are aimed at preventing adverse outcomes, including stroke, such intensified management may inadvertently increase the risk of stroke.

Crucial variables such as atrial fibrillation were reported only in one study, and their corresponding CHADS-VASc scores were not reported in any of the studies, which may lead to variations in the stroke risk between cohorts. The use of multiple blood pressure medications and different routes of administration, along with variable definitions of “intensified” and “non-intensified” groups and their patients, together with the lack of randomized trials, appears to significantly contribute to the moderate heterogeneity of results, which was corroborated in our sensitivity analysis.

### 4.3 AKI

AKI is a common clinical event that affects up to 15% of hospitalized patients (20). A total of 20%–50% of AKI patients develop progressive CKD, while 3%–15% reach end-stage kidney disease, all associated with increased mortality (21). Hypertension and hypotension are well-known risk factors for AKI; however, BP variability may also contribute. Our finding of increased AKI risk (OR 1.22) may reflect not only hypotension but also BP variability, as supported by perioperative studies demonstrating AKI risk from fluctuations in SBP independent of mean BP (22). Another study in patients undergoing surgery for acute aortic dissection showed that patients with AKI had higher SBP variability (23). Underlying chronic kidney disease (CKD) is a known risk factor for AKI (24); however, it is unlikely to account for a higher AKI rate in the intensified BP control group because of a similar rate of CKD in both groups. One included study had a high proportion of UTI admissions—a known AKI risk factor (4, 25). However, both groups had similar UTI rates, and the exclusion of this study did not alter the pooled AKI estimate.

### 4.4 Myocardial infarction

In contrast to the pooled analysis, the sensitivity analysis limited to in-hospital BP intensification showed significantly increased MI risk. This discrepancy may be due to the use of intravenous antihypertensive medications during hospitalization, which can cause abrupt reductions in perfusion pressure. The shorter follow-up in the sensitivity analysis may capture early adverse events from such interventions, while the longer follow-up in the pooled analysis may dilute these effects by reflecting overall cardiovascular risk. However, the presence of only two studies in the sensitivity analysis and high heterogeneity warrant careful interpretation of these results. Despite high statistical heterogeneity (e.g., *I*^2^ = 94% for myocardial infarction and 100% for length of stay), quantitative pooling was retained to provide an overall estimate under a random-effects model. The decision to pool was based on the clinical and methodological comparability of included studies; each study examined intensified vs. standard blood pressure management in non-cardiac hospitalizations using similar definitions of exposure and outcomes. Furthermore, directionality of effects was largely consistent across studies, suggesting a shared underlying effect despite differences in magnitude. Quantitative synthesis thus complements the narrative summary by providing a global measure of association, while acknowledging uncertainty due to heterogeneity.

### 4.5 Strengths and limitation

The findings from the current study should be interpreted as hypothesis-generating within the context of its limitations. First, all included studies were observational despite adjustment using propensity score matching; the individual patient risk for stroke and AKI was not considered. Second, there were mild variations in the definition of intensified and non-intensified blood pressure across the studies, despite the same principle: patients received more antihypertensive medication in the intensified group compared with the conservative management group. Our analysis is limited by significant heterogeneity, potentially due to the use of various antihypertensive classes and administration routes. Potential confounders may be the more frequent IV administration route and more frequent hydralazine use in the intensified BP control group. These factors highlight the need for further randomized studies to answer these clinical questions. All studies included only older adults, with a mean age of approximately 70; thus, our findings are not generalizable to younger populations.

A notable methodological heterogeneity among the included studies relates to the timing of blood pressure (BP) intensification. In their study, Anderson et al. (4) initiated antihypertensive intensification only at hospital discharge, whereas the remaining studies (8, 26, 27) implemented treatment adjustments during the inpatient stay. This distinction carries clinical significance, as in-hospital intensification exposes patients to the acute hemodynamic fluctuations and autoregulatory challenges associated with the hospital environment—factors that are largely absent when therapy is initiated at discharge. Consequently the Anderson et al. (4) study primarily reflects outpatient hemodynamic adaptation rather than true inpatient exposure. When this study was excluded in a subgroup analysis, the direction and magnitude of associations for stroke, AKI, and MI remained consistent with the overall pooled estimates, suggesting that the main findings were robust and not driven by timing-related differences. Nonetheless, these results highlight the need for future investigations to distinguish between inpatient and discharge-only BP management, as these represent distinct clinical interventions with potentially divergent risk profiles.

Future research should focus on prospective, randomized controlled trials designed specifically to evaluate the safety and effectiveness of intensified BP management in the inpatient setting. Such trials should be stratified by the timing of intervention—that is, whether blood pressure intensification occurs during hospitalization or is initiated at discharge, as the pathophysiological and clinical contexts may differ substantially. Incorporating standardized BP targets, consistent adverse event definitions, and follow-up into the postdischarge period will be essential. In addition, future studies should address modifiable inpatient contributors to transient BP elevation, such as anxiety, pain, medication reconciliation, and iatrogenic fluid shifts, to better inform intervention thresholds and avoid overtreatment.

## 6 Conclusion

In this meta-analysis of patients with non-cardiac admissions, our findings suggest that intensified blood pressure control during admission and on discharge was associated with significantly higher odds of stroke, AKI, and prolonged length of stay. The increased chance of MI was not statistically significant. These findings, derived exclusively from retrospective observational cohorts, underscore the potential harm of intensified blood pressure control and highlight the urgent need for prospective, randomized inpatient BP trials, particularly those comparing interventions initiated during hospitalization vs. at discharge to establish causal relationships and optimize inpatient hypertension management.

## Data Availability

Publicly available datasets were analyzed in this study. The data can be found here: https://doi.org/10.1001/jamainternmed.2019.3007; https://doi.org/10.1001/jamainternmed.2020.7501; https://doi.org/10.1161/HYPERTENSIONAHA.121.17279; https://doi.org/10.1001/jamainternmed.2024.6213.
