# Supplementary material for: Intensified blood pressure control during hospital admission and on discharge: a systematic review and meta-analysis of retrospective cohort studies
Source: Front Cardiovasc Med. 2026 Feb 5;13:1691926. doi: 10.3389/fcvm.2026.1691926 (PMC12916387; doi:10.3389/fcvm.2026.1691926)
Supplement: Supplementary file 1 [file Supplementaryfile1.docx]

**
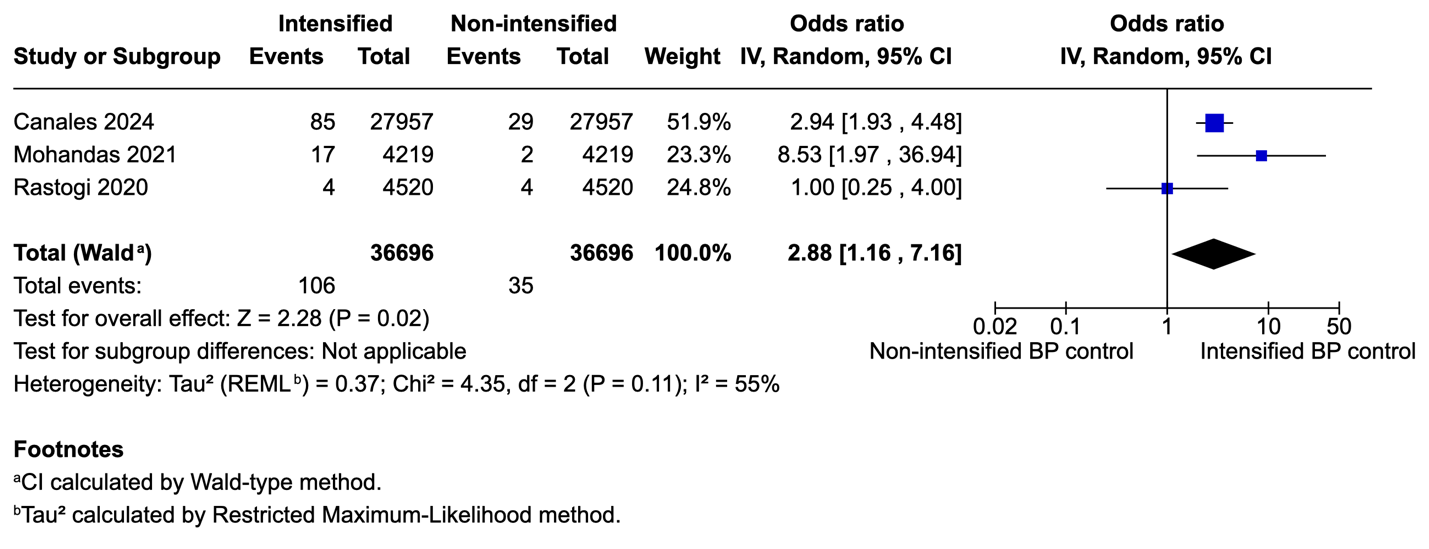
**

**Supplementary Figure 1.**

**
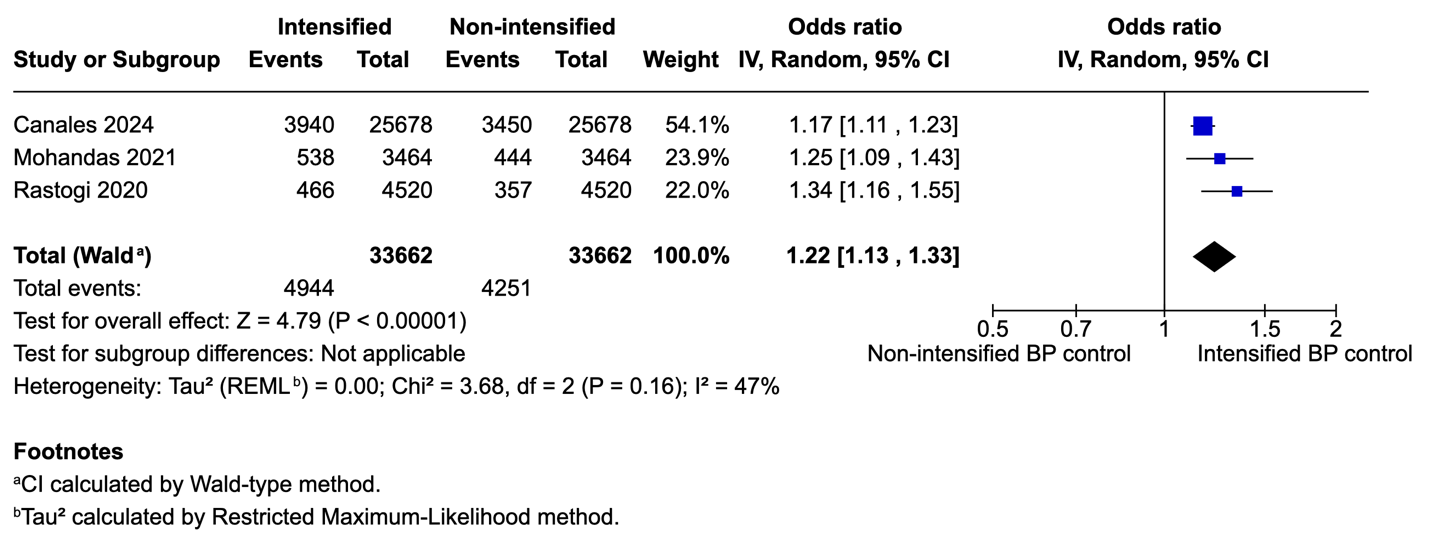
**

**Supplementary Figure 2.**

**
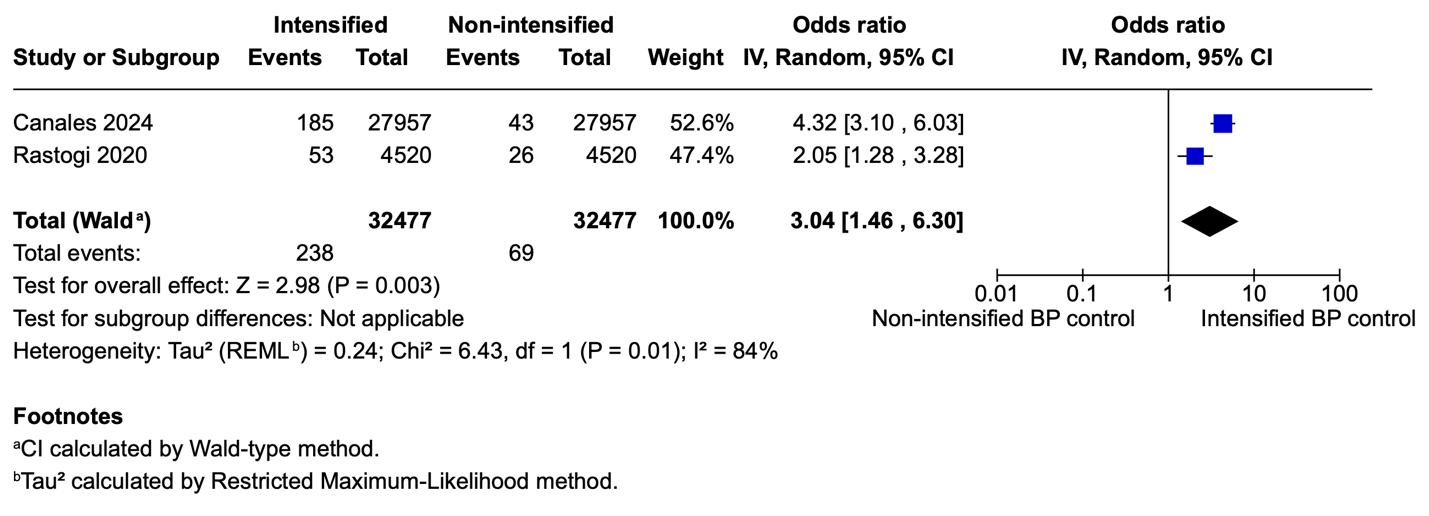
**

**Supplementary Figure 3.**

**
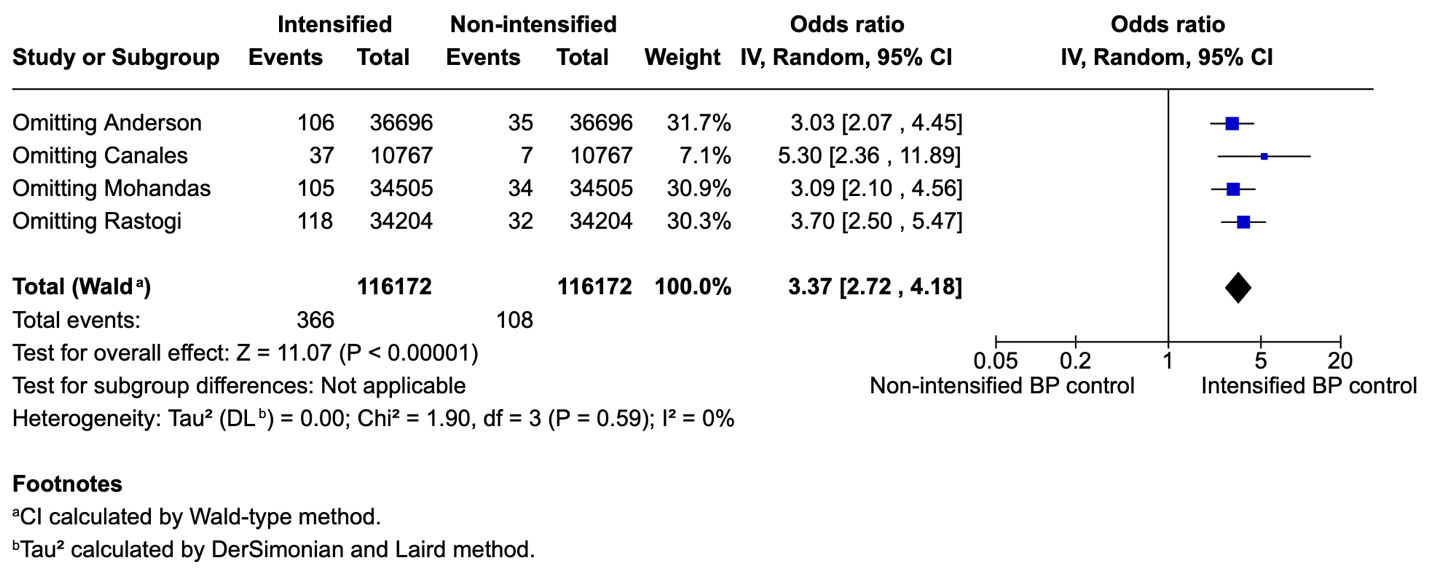
**

**Supplementary Figure 4.**

**
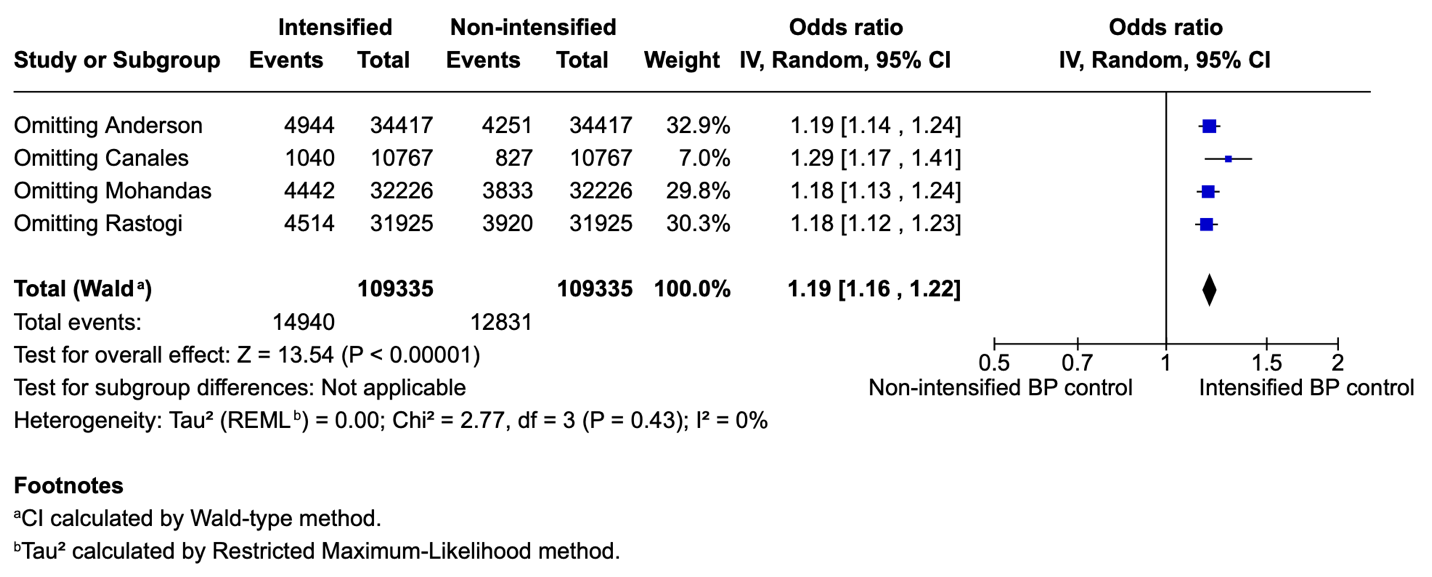
**

**Supplementary Figure 5.**


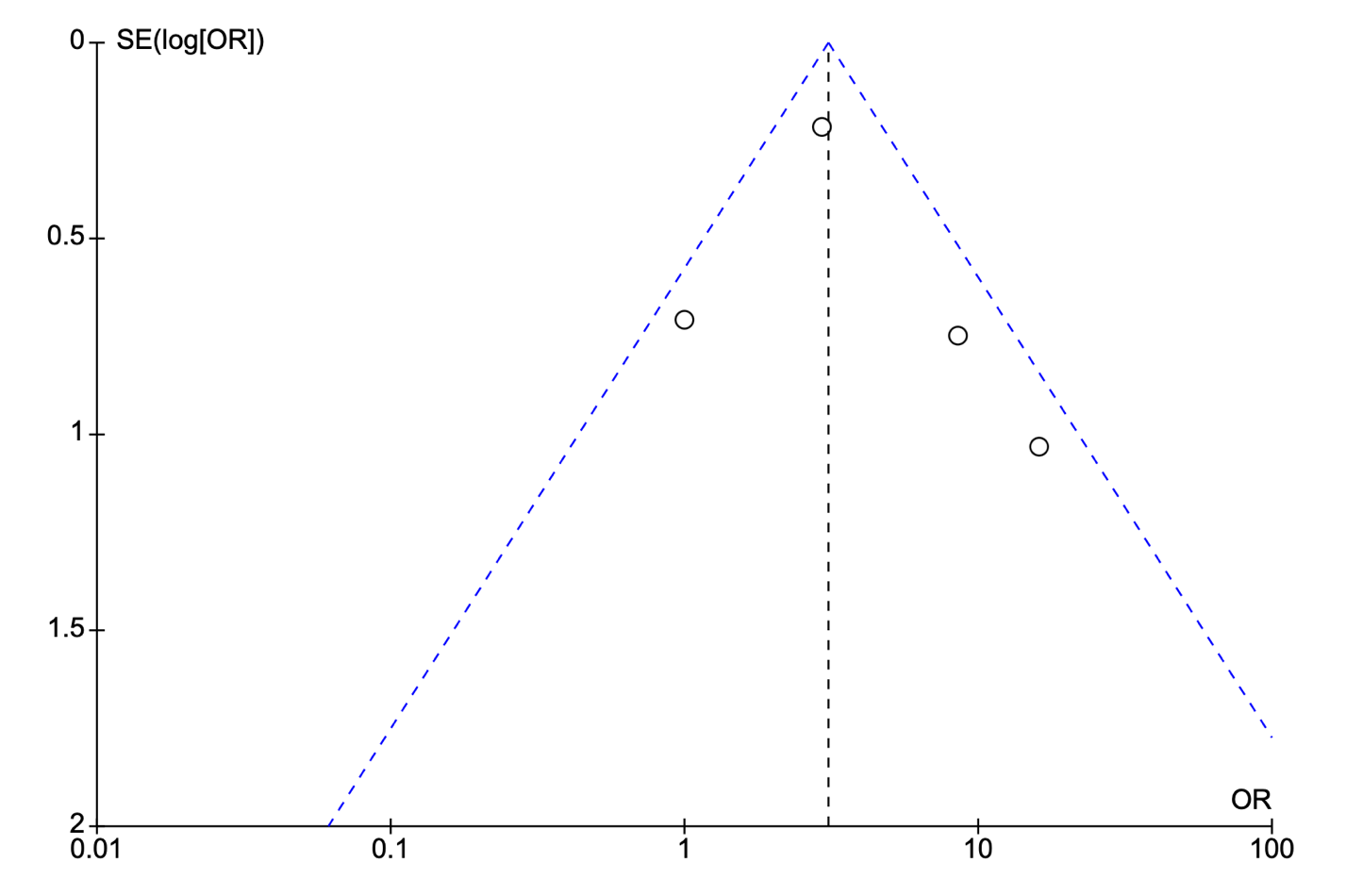


**Supplementary figure 6.**

**Supplementary Table 1.** Critical appraisal of individual studies according to the Cochrane Collaboration’s tool for assessing risk of bias in non-randomized trials.

**Risk of bias summary for non-randomized studies (ROBINS-I)**

| Study | Bias due to confounding | Bias in selection of participants | Bias in classification of interventions | Bias due to deviations from intended interventions | Bias due to missing data | Bias in measurement of outcomes | Bias in selection of the reported result | Overall risk of bias judgment |
| --- | --- | --- | --- | --- | --- | --- | --- | --- |
| Anderson | Moderate | Low | Low | Low | Low | Low | Low | Low |
| Rastogi | Moderate | Low | Low | Low | Low | Low | Low | Low |
| Mohandas | Moderate | Low | Low | Low | Low | Low | Low | Low |
| Canales | Moderate | Low | Low | Low | Low | Low | Low | Low |

**Supplementary Table 1. Risk of bias assessment, ROBINS-I**

**Supplementary Figure 1**. Forest plot of sensitivity analysis restricted to studies with BP

intensification during hospitalization only, showing odds of stroke. Each study is represented bya blue square proportional to its weight, with horizontal lines denoting 95% confidence intervals (CIs). The pooled estimate (black diamond) was calculated using a random-effects model(Restricted Maximum-Likelihood method) with CIs determined by the Wald-type approach. Overall, intensified BP control was associated with significantly higher odds of stroke compared with non-intensified control (pooled OR = 2.88, 95% CI 1.16–7.16; P = 0.02). Moderate between-study heterogeneity was observed (I² = 55%, P = 0.11). Abbreviations: BP – blood pressure; CI – confidence interval; IV – inverse variance; OR – odds ratio; REML – restricted maximum-likelihood; Wald – Wald-type method.

**Supplementary Figure 2.** Forest plot of sensitivity analysis restricted to studies with BP

intensification during hospitalization only, showing chance of AKI. Each study is depicted by a blue square proportional to its statistical weight, with horizontal lines representing 95% confidence intervals (CIs). The pooled odds ratio (black diamond) was calculated using a random-effects model (Restricted Maximum-Likelihood method) with Wald-type CIs. Across three retrospective cohort studies, intensified BP control was associated with a statistically significant increase in the odds of adverse events compared with non-intensified control (pooled OR = 1.22, 95% CI 1.13–1.33; P < 0.00001). Between-study heterogeneity was moderate (I² =47%, P = 0.16). Abbreviations: AKI – acute kidney injury; BP – blood pressure; CI – confidence interval; IV – inverse variance; OR – odds ratio; REML – restricted maximum-likelihood; Wald – Wald-type method.

**Supplementary Figure 3.** Forest plot of sensitivity analysis restricted to studies with BP intensification during hospitalization only, showing odds of myocardial infarction. Each study is represented by a blue square proportional to its relative weight, and horizontal lines indicate 95% confidence intervals (CIs). The pooled odds ratio (black diamond) was calculated using a random-effects model (Restricted Maximum-Likelihood method) with CIs derived via the Wald- type approach. Intensified BP control was associated with significantly higher odds of MI events compared with non-intensified control (pooled OR = 3.04, 95% CI 1.46–6.30; P = 0.003). Substantial between-study heterogeneity was observed (I² = 84%, P = 0.01). Abbreviations: BP – blood pressure; CI – confidence interval; IV – inverse variance; MI – myocardial infarction; OR – odds ratio; REML – restricted maximum-likelihood; Wald – Wald-type method.

**Supplementary Figure 4.** Forest plot showing leave-one-out sensitivity analysis of stroke odds comparing intensified versus non-intensified inpatient blood pressure (BP) control strategies. Each line represents the pooled odds ratio (OR) recalculated after sequential omission of one study, with blue squares indicating study-specific estimates and horizontal lines showing 95% confidence intervals (CIs). The pooled estimate (black diamond) was derived using a random-effects model (Restricted Maximum-Likelihood method) with Wald-type CIs. The overall association remained robust across all iterations, showing consistently higher odds of adverse events with intensified BP control (pooled OR = 3.37, 95% CI 2.72–4.18; P < 0.00001). No heterogeneity was observed (I² = 0%, P = 0.59), confirming the stability of the results. Abbreviations: BP – blood pressure; CI – confidence interval; IV – inverse variance; OR – odds ratio; REML – restricted maximum-likelihood; Wald – Wald-type method.

**Supplementary Figure 5.** Forest plot showing leave-one-out sensitivity analysis of AKI odds comparing intensified versus non-intensified inpatient blood pressure (BP) control strategies. Each line represents the pooled odds ratio (OR) recalculated after sequential exclusion of one study, with blue squares denoting study-specific estimates and horizontal lines showing 95% confidence intervals (CIs). The pooled estimate (black diamond) was obtained using a random-effects model (Restricted Maximum-Likelihood method) with Wald-type CIs. The association between intensified BP control and adverse events remained consistent across all analyses (pooled OR = 1.19, 95% CI 1.16–1.22; P < 0.00001), indicating high robustness of the overall findings. No heterogeneity was observed (I² = 0%, P = 0.43). Abbreviations: AKI – acute kidney injury; BP – blood pressure; CI – confidence interval; IV – inverse variance; OR – odds ratio; REML – restricted maximum-likelihood; Wald – Wald-type method.

**Supplementary Figure 6.** Funnel plot assessing publication bias for risk of stroke in studies comparing intensified versus non-intensified inpatient blood pressure (BP) control. Each circle represents an individual study plotted by its effect size [log odds ratio (OR)] on the x-axis and its standard error [SE(log OR)] on the y-axis. The vertical dashed line indicates the pooled effect estimate, and the blue dashed lines represent the expected 95% confidence limits around the summary effect. The symmetrical distribution of studies around the pooled estimate suggests no substantial evidence of publication bias. Abbreviations*:* OR = odds ratio; SE = standard error.
